# The effectiveness of the neonatal diagnosis-related group scheme

**DOI:** 10.1371/journal.pone.0236695

**Published:** 2020-08-12

**Authors:** Marcello Montefiori, Michela Pasquarella, Paolo Petralia

**Affiliations:** 1 Department of Economics, University of Genoa, Genoa, Italy; 2 IRCCS Gaslini Children’s Hospital, Genoa, Italy; Murdoch University, AUSTRALIA

## Abstract

The goal of this study is to investigate the effectiveness of the neonatal diagnosis-related group scheme in patients affected by respiratory distress syndrome. The variable costs of individual patients in the same group are examined. This study uses the data of infants (N = 243) hospitalized in the Neonatal Intensive Care Unit of the Gaslini Children’s Hospital in Italy in 2016. The care unit’s operating and management costs are employed to estimate the average cost per patient. Operating costs include those related to personnel, drugs, medical supplies, treatment tools, examinations, radiology, and laboratory services. Management costs relate to administration, maintenance, and depreciation cost of medical equipment. Cluster analysis and Tobit regression are employed, allowing for the assessment of the total cost per patient per day taking into account the main cost determinants: birth weight, gestational age, and discharge status. The findings highlight great variability in the costs for patients in the same diagnosis-related group, ranging from a minimum of €267 to a maximum of €265,669. This suggests the inefficiency of the diagnosis-related group system. Patients with very low birth weight incurred costs approximately twice the reimbursement set by the policy; a loss of €36,420 is estimated for every surviving baby with a birth weight lower than 1,170 grams. On the contrary, at term, newborns cost about €20,000 less than the diagnosis-related group reimbursement. The actual system benefits hospitals that mainly treat term infants with respiratory distress syndrome and penalizes hospitals taking care of very low birth weight patients. As a result, strategic behavior and “up-coding” might occur. We conduct a cluster analysis that suggests a birth weight adjustment to determine new fees that would be fairer than the current costs.

## Introduction

Diagnosis-related group (DRG)-based prospective payment schemes are commonly adopted in several national healthcare systems. The literature agrees that DRGs are effective in controlling the prices of medical services and that they incentivize cost containment goals [1]. Hospital revenues depend on DRG prices that in turn, directly affect the financial risk borne by the hospital [2]. The cost depends on many variables that rely both on patients’ conditions and the size, ownership (public, private) [3], and organization (teaching or research hospital) [4–7] of the hospital. With DRG, patients with similar diagnoses and homogenous resource consumption are grouped together and reimbursed in a similar manner [8, 9]. Diagnosis-related group reimbursement schemes are considered effective if the price is fairly defined and accurately reflects the costs incurred by the hospital for patient treatment and stay [10, 1]. Length of stay (LOS) is generally considered to be one of the most relevant indicators of hospital activity and resource consumption [11, 12]. As a result, a performant DRG system should be able to predict resource consumption for patients belonging to a specific DRG, mainly by considering the expected LOS [13]. If the DRG reimbursement was not correctly defined, favorable conditions for opportunistic behavior may occur. Attention should be placed on the variation of patient severity and costs within a group. According to [14], the risk lies in the adoption of one of the following strategies: "creaming, the over-provision of services to low cost patients; skimping, the under-provision of services to high cost patients; dumping, the explicit avoidance of high cost patients".

Considering neonatal diagnosis, DRGs seem to fail in “matching” the actual resource utilization. This is mainly due to the high LOS variability between groups [15–19], which directly correlates to the gestational age and weight at birth [20–25]. It is estimated that costs for preterm babies are 25 times greater than for “in-terms” and those with no complications [20]. In particular, the cost of Very Low Birth Weight (VLBW) babies, that is, infants with complications at birth and birth weights lower than 1,500 grams, can vary between €38,660 and €116,180 based on analysis conducted in the US, England, Finland, and Sweden [22].

Respiratory Distress Syndrome (RDS) is one of the most frequent complications in preterm infants [26] and is attributable to a deficiency of pulmonary surfactant in premature neonates [27]. The risk of contracting RDS decreases significantly with gestational age [28, 29]. The management of RDS involves prolonged artificial respiratory support and the use of expensive therapy [30]. For example, surfactant replacement helps maximize the surface area available for gas exchange in the lungs while a lack thereof causes breathing difficulties. Hence, surfactant therapy is commonly used for babies with RDS.

In the Italian health-care system, the hospitalization of babies born with RDS fall under DRG 385, “neonates, deceased or transferred to another acute care facility,” and DRG 386, “extreme immaturity or respiratory distress syndrome, neonate". These codes match the US Medicare DRG codes of 789 and 790, respectively. Infants who do not survive after birth or are transferred to another hospital for the continuation of care, independent of disease type, are included in DRG 385. Diagnosis-related group 386 includes infants with a birth weight less than 1,000 grams and RDS patients regardless of whether they are preterm. Hence, a patient’s discharge status becomes an important discriminant affecting DRG assignment.

In Italy, regions have been autonomous in the organization and management of healthcare, including the definition of the DRG tariffs, since the late 90s. Therefore, local regional governments can opt to adopt the national DRG tariffs or set new tariffs. In the region of Liguria, DRGs 385 and 386 show the same national tariffs. However, it should be noted that, with reference to Gaslini Children’s Hospital, a 20% increase is assigned: DRGs 385 and 386 are therefore reimbursed €6,522 and €36,866, respectively. Moreover, the Italian healthcare system provides a threshold value for each DRG, which indicates the maximum number of hospitalization days, beyond which an additional daily reimbursement is assigned to the hospital. The threshold value for DRGs 386 and 385 is equal to 135 days and 4 days, respectively. The hospital receives an additional reimbursement on a daily basis equal to € 354 and € 84, respectively, per day of hospitalization exceeding this threshold. However, this marginal cost sharing does not even fully cover per day hospitalization costs. For this reason, we do not believe that any incentive to strategic behavior could be provided by this additional (marginal) cost reimbursement.

The present study aims at estimating the actual costs incurred for the hospitalization of extremely immature babies or infants affected by RDS. For this purpose, DRGs 385 and 386 patients, hospitalized at Gaslini Children’s Hospital in 2016, were analyzed using clinical and administrative records.

## Methods

The study is carried out as part of routine checks conducted in the Gaslini Children’s Hospital, and thus, ethical approval is not required. As with all studies conducted in the hospital environment, hospital management approved the study’s protocols. Management was responsible for ensuring the ethical aspects of all hospital activities as the entire study was organized in conjunction with Gaslini teams. Upon entering the hospital, all patients (or parents) sign an informed consent form regarding hospital treatment and its terms and conditions. The research was carried out in full accordance with the Italian law on privacy and all data were appropriately pseudonymized by Gaslini Children’s Hospital by associating each patient with a numerical code.

Gaslini Children’s Hospital is located in Genoa, a city in Northwest Italy. It is a referral hospital for many disciplines such as general practice, neonatal, oncology, and orthopedic surgery. Since 1959, it has been formally recognized as a scientific institute for research, hospitalization, and healthcare by the Italian Ministry of Health, with about 30,000 hospitalizations and 550,000 outpatient services per year. During 2016, the hospital accepted more than 70% of regional hospitalizations with the 769 ICD-9-CM code. Furthermore, about 80% of the overall regional discharges with DRG 386 were made by Gaslini Children’s Hospital’s Neonatal Intensive Care Unit (NICU). The research data is based on 243 infants hospitalized in the hospital’s NICU from 1 January to 31 December 2016. Using the International Classification of Disease, patients with the principal or secondary diagnosis code 769, “respiratory distress syndrome in new-born,” were included in the study sample. This diagnosis code is annotated in the “Hospital Discharge Form.” In particular, infants discharged with DRG 386 were tagged with the 769 code as the principal or secondary diagnosis. A subset of 219 cases were discharged with DRG 386, while 24 cases died or were transferred to another hospital and discharged with DRG 385.

The operating and management costs used in the present analysis were provided by the NICU. These included personnel costs (physicians and staff); utilities (electricity, water, gas, telephone); medical supplies and drugs costs; treatment tools; management costs (administration, maintenance, depreciation cost of medical equipment); and department overhead costs (cleaning, mortgages, kitchen and laundry, other). These costs were obtained from the department’s annual balance sheet and distributed among patients on a daily basis. Examinations, radiology, laboratory services, and other patient-specific direct costs were analytically imputed to each patient [31]. Hence, assessing the total cost on a daily basis and taking into account care unit capacity, the mean hospital per day cost was estimated. Moreover, LOS, number of examinations, discharge status, and the neonatal characteristics of the infants were also considered in the analysis. By jointly considering the mean hospital cost, patients’ LOS, and regional DRG tariffs, the inpatient reimbursement adequacy was investigated.

The K-means clustering method was implemented to create a homogeneous group of patients [32]. Given that this method requires pre-determining the number of clusters, a dendrogram and the Within Group Sum of Square (WSS) error plot were used to identify the appropriate number of groups. The sample was studied before and after clustering. The variables included in the Cluster Analysis relate to the DRG type, birth weight, gestational age, number of examinations, gender, type of discharge (home, died, or transferred to another hospital), and place of birth (“inborn” if the infant was born at Gaslini Children’s hospital or “outborn” if born elsewhere). It should be noted that birth weight is a variable that cannot be affected by biased recovery policies. For each cluster, the mean cost was estimated, taking into account the assistance and examination costs for each patient included in the group. The results obtained were used to define new fees strictly related to the actual costs incurred by the hospital in treating these patients.

Tobit regression models were used to capture the relationships between the costs and determinants of a newborn’s recovery. The LOS was used as a proxy for costs in order to avoid discretionary measures. The Tobit model is used, provided that LOS (the dependent variable in the regression) is left censored at zero [33]. By comparing two Tobit models, we examined the influence of neonatal and clinical characteristics on LOS. Both models use the same set of infant characteristics such as gestational age, number of examinations, discharge status, gender, and place of birth to predict the LOS; however, the latter also considers the VLBW variable to investigate the economic impact of babies with birth weights lower than 1,500 grams on LOS. The models take the following general form:

$$y_{i}^{*}=\beta_{0}+\sum_{k=1}^{K}\beta_{k}x_{i}+\varepsilon_{i}\;\varepsilon_{i}\sim N(0,\sigma^{2})$$ (1)

yi={yi*,ifyi*>00,ifyi*≤0i=1,2….N (2)

where $y_{i}^{*}$ and *y*_*i*_ represent patient *i’s* unobservable and observed LOS, respectively. If the observed LOS is 0 (under 24 hours), the observation is censored. *x*_*i*_ is the regressor, which will be discussed in detail in Eqs (1) and (2); *β*_*k*_ (k = 1,2…N) represents the estimable coefficients of the model, *β*_*o*_ is the constant, and *ε*_*i*_ denotes the standard random error term. The first and the second model can be represented by Eqs (3) and (4), respectively:

$${LOS}_{i}=\beta_{0}+\beta_{1}{GA}_{i}+\beta_{2}D_{i}+\beta_{3}B_{i}+\beta_{4}G_{i}+\beta_{5}N_{i}+\varepsilon_{i}$$ (3)

$${LOS}_{i}=\beta_{0}+\beta_{1}{GA}_{i}+\beta_{2}D_{i}+\beta_{3}B_{i}+\beta_{4}G_{i}+\beta_{5}N_{i}+\beta_{6}{VLBW}_{i}+\varepsilon_{i}$$ (4)

where “GA” is the gestational age, “N” is the number of examinations, while “D,” “B,” and “G” denote the dummy variables of the model. “D” represents the type of discharge; it is 1 if the patient died, and 0 otherwise. “G” is the gender of the baby and is 1 if male and 0 if female. “B” is the place of birth, which is 1 if the infant is outborn and 0 if inborn. The second model differs from the first for the “VLBW” variable, which is a dummy that takes a value of 1 if the baby’s birth weight is lower than 1,500 grams and 0 otherwise.

## Results

**Fig 1:**
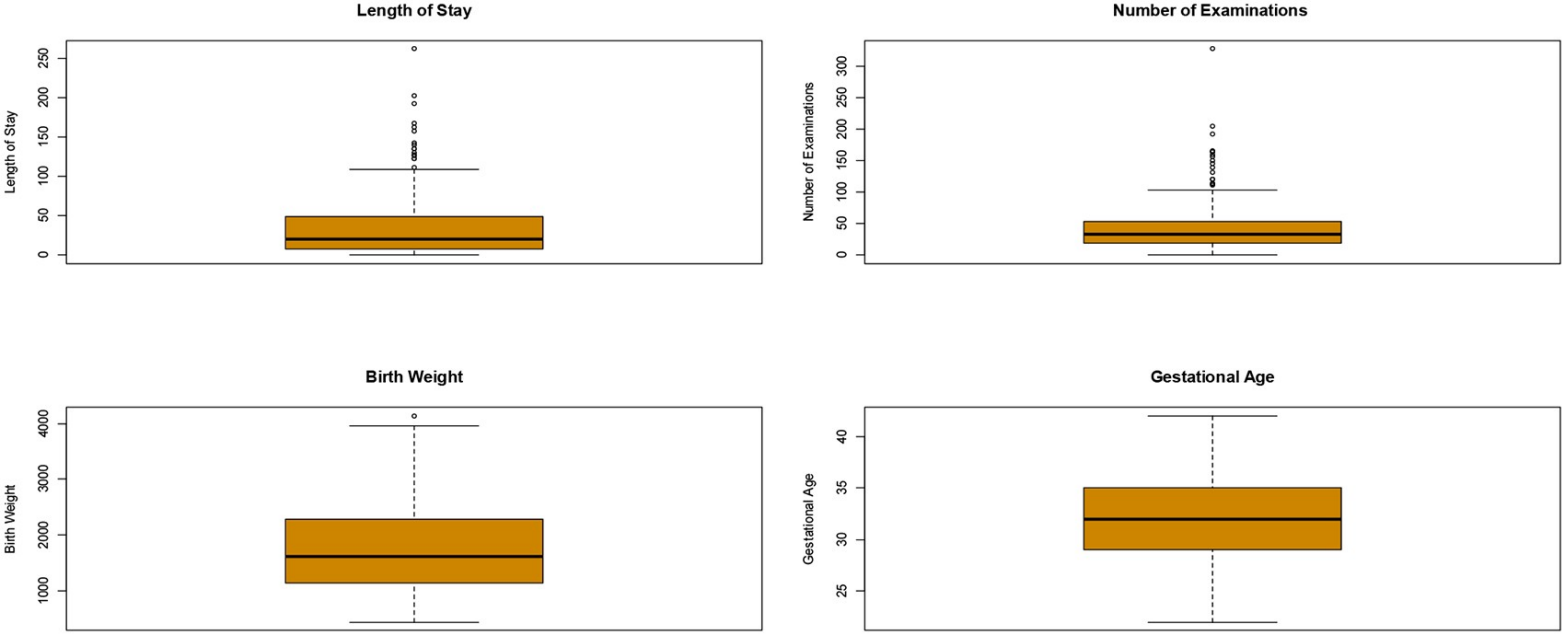
Sample variability.

The mean per-day hospital cost in the NICU is estimated to be €820. This value is obtained by the analysis of total cost reassignment on a daily basis and only considers the total number of beds in the care unit. Considering the different DRG types and their relative resource utilization, we estimate a mean hospitalization cost of €2,168 and €39,419 for DRGs 385 and 386, respectively. Compared to the reimbursement, this results in a mean gain of €4,354 for each patient discharged with DRG 385 and a mean loss of €2,553 for each patient discharged with DRG 386. Fig 1 highlights the great sample variability in the LOS, number of examinations, birth weight, and gestational age. The LOS ranges from 0 (under 24 hours) to 262 days, number of examinations from 0 to 328, and birth weight from 435 grams to 4,140 grams with a mean of 1,781. The gestational age distribution is symmetric with the mean value (32) matching the median and the whiskers of the boxplot have a similar length.

The LOS positively correlates with the number of examinations, and negatively with birth weight and gestational age. The number of examinations tends to increase if the infant has a low birth weight or low gestational age. Birth weight strongly positively correlates with gestational age. Conversely, no correlation occurs between the number of examinations and the birth weight and gestational age.

**Fig 2:**
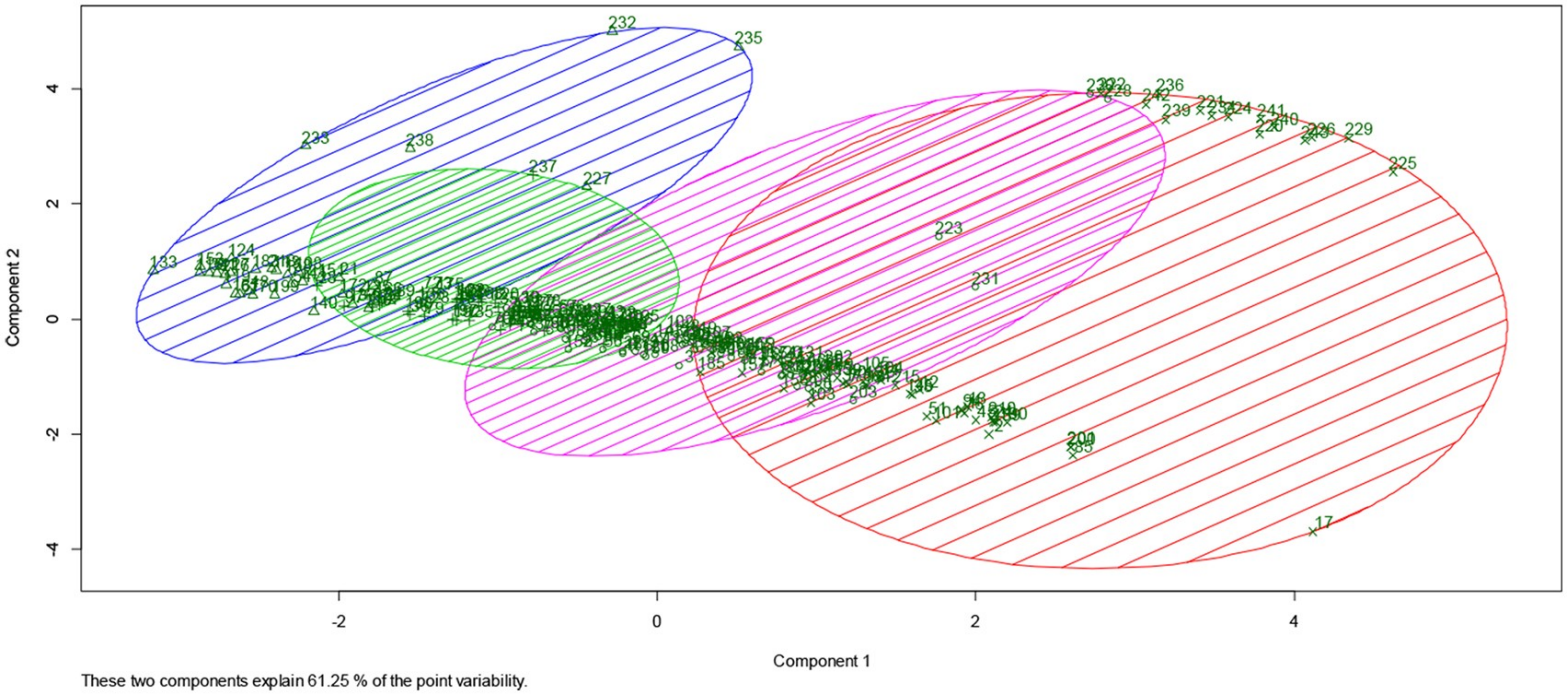
Cluster analysis.

Fig 2 reports the cluster analysis output. The green dots represent the observations while the components along the axis reflect the dimensions with the largest variance, which explain 61.25% of the total variability [34]. These components are generated by the Principal Component Analysis, which reduces dimensional data to plot the clusters in a two-dimensional space [34]. The value of the first component changes continuously in each cluster, while the second component’s value has discontinuous jumps, particularly for the red and blue clusters.

Dots at the top of the plot represent patients discharged with DRG 385; the ones located higher died while the ones located at the average height were transferred to another hospital. Dots at the bottom refer to infants discharged with DRG 386. The amplitude of the circles indicates how similar patients are between groups. Cluster 2, in green, is the smallest and does not include deceased patients. Its dots are very close to one another, highlighting that this cluster has the lowest variability. On the contrary, Cluster 4, in red, is the biggest and, in fact, presents a greater variability within its group. It is comprised of patients with low birth weights and infants discharged with DRG 385 who died after birth. Therefore, it is quite a homogenous group from a neonatal characteristics’ perspective, but heterogeneous when looking at the status of discharge.

Table 1 summarizes the characteristics and descriptive statistics of each cluster. From the table, we can observe that Clusters 1 and 2 do not include VLBW newborns, but include at term infants, contrary to Clusters 3 and 4. In particular, Cluster 4 includes only VLBW patients with birth weights lower than 1,170, which accounts for 78% of all deceased babies in the sample discharged with DRG 385. It excludes patients transferred to other hospitals after birth. Moreover, 75% of the patients in this cluster have a gestational age less than 28 weeks. The mean hospitalization cost is €73,286, ranging from €268 to €265,669. Cluster 3 includes only preterm infants with a birth weight lower than 1,885 grams and a mean gestational age of 31 weeks. The VLBWs in this cluster have birth weights greater than 1,190 grams. The estimated mean hospitalization cost is €32,364 and ranges from €562 to €126,997. Cluster 2 includes only surviving babies with a birth weight between 1,920 grams and 2,800 grams and a mean gestational age of 34 weeks. Patients in this cluster have the lowest number of examinations. The mean hospitalization cost varies between €1,407 and €44,842, with an estimated mean value of €12,177. Finally, Cluster 1 mainly includes infants born at-term; its mean gestational age is 39 weeks and birth weight ranges between 2,870 grams and 4,140 grams. The mean hospitalization cost is €10,899 and ranges between €516 and €35,374.

**Table 1:** Cluster characteristics.

		***Cluster 1***	***Cluster 2***	***Cluster 3***	***Cluster 4***	
***Number of observations***		34	57	86	66	
***Died***		2 [11%]*	0	2 [11%]	14 [78%]	
***Transferred***		3 [50%]	1 [17%]	2 [33%]	0	
***Outborn***		17 [24%]	20 [28%]	27 [38%]	7 [10%]	
***Male***		22 [17%]	36 [28%]	47 [37%]	22 [17%]	
***VLBW***		0	0	47 [42%]	66 [58%]	
***Preterm infants (GA < 37)***		5 [2%]	54 [26%]	86 [41%]	66 [31%]	
***At term infants***		29 [91%]	3 [9%]	0	0	
	**Min**.	**1**^**st**^ **Q**	**Median**	**Mean**	**3**^**rd**^ **Q**	**Max**
***Cluster 1***						
***Cost of hospitalization***	€516	€4,998	€10,306	€10,899	€15,774	€35,374
**Length of Stay**	1	5.25	10.5	11.03	16	45
**Number of Examinations**	3	19.25	28.5	31.03	43.5	68
**Birth Weight**	2,870	3,125	3,360	3,373	3,572	4,140
**Gestational Age**	35	37	39	39	40	42
***Cluster 2***						
***Cost of hospitalization***	€1,407	€7,134	€9,548	€12,177	€15,186	€44,842
**Length of Stay**	2	7	9	12.05	15	44
**Number of Examinations**	6	13	18	23.46	30	63
**Birth Weight**	1,920	2,080	2,280	2,294	2,440	2,800
**Gestational Age**	30	33	34	34	35	38
***Cluster 3***						
***Cost of hospitalization***	€562	€13,667	€30,465	€32,364	€42,295	€126,997
**Length of Stay**	1	15.25	31	33.64	42.75	126
**Number of Examinations**	2	22.25	30.5	40.66	48.75	205
**Birth Weight**	1,190	1,342	1,495	1,510	1,690	1,885
**Gestational Age**	27	30	31	31	32	36
***Cluster 4***						
***Cost of hospitalization***	€267	€40,997	€67,673	€73,286	€107,420	€265,669
**Length of Stay**	0	44.5	68	75.73	108	262
**Number of Examinations**	0	46	59.5	75.45	97	328
**Birth Weight**	435	785	894	870	1,020	1,170
**Gestational Age**	22	26	27	27	28	34

* All percentages are calculated with respect to the number of observations in the sample.

**Fig 3:**
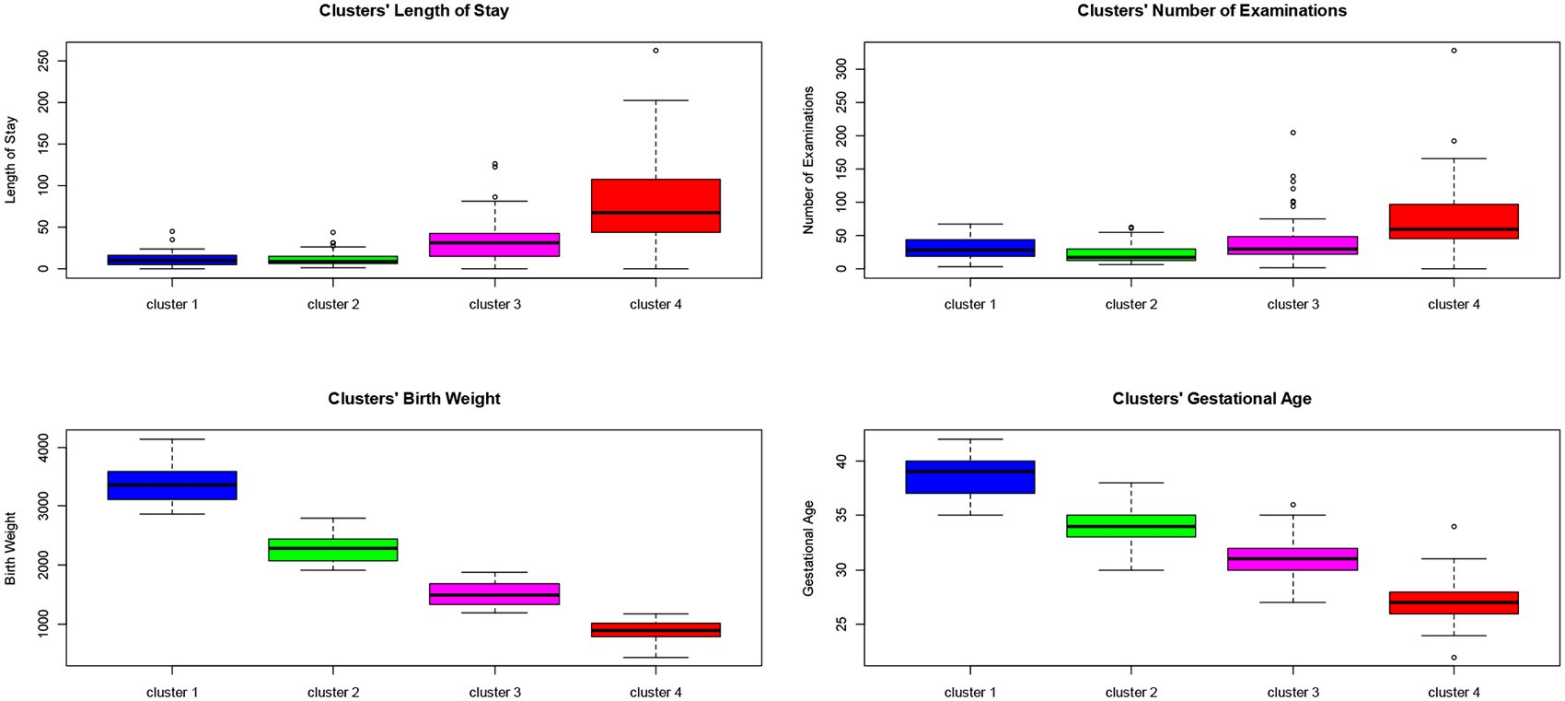
Within group variability.

As shown in Fig 3, birth weight is the main cluster discriminant. In fact, there are no overlaps amongst the groups. The black bold horizontal line in the boxplots emphasizes that infants with the lowest birth weights are characterized, on average, by the highest LOS and number of examinations. Nonetheless, the height of the red box indicates that low birth weight infants present the greatest variability. The patients with a greater birth weight/gestational age have less variability in their LOS and the number of examinations as reported in the green and blue box plots.

Results from the Tobit regression are reported for each model in Table 2, with the corresponding 95% confidence interval plots. In both cases, LOS is the dependent variable. In order to run the regression correctly, we deleted observations with an LOS less than 1 (i.e., those infants who died within 24 hours) from the sample, resulting in 242 observations.

**Table 2:** Tobit regressions results for models 1 and 2.

	Model 1		Model 2	
	Coefficient	Std. Err.	Coefficient	Std. Err.
Use the "Insert Citation" button to add citations to this document.	-3.39***	0.37	-2.22***	0.47
Use the "Insert Citation" button to add citations to this document.				
**GA**				
**Type_of_discharge**	-35.78***	5.32	-38.13***	5.19
**Place_of_birth**	-5.94*	3.04	-5.43*	2.95
**Gender**	-9.15***	2.69	-7.24***	2.66
**N_of_examinations**	0.62***	0.04	0.6***	0.04
**VLBW**			15.12***	3.89
**Cons**	126.28***	12.82	82.01***	1.69

In the first model, all variables are significant at the 1% significance level except the place of birth (significant at 10%). The type of discharge and gestational age has a great impact on LOS. A new-born who dies or who was born at-term has a significantly lower LOS than a surviving infant or a late preterm. Males have lower LOS than females. The number of examinations increases with LOS.

In the second model, we notice that the VLBW variable has a significant (1% level) impact on LOS. Additionally, the impact of the patient’s death slightly increases, while the impact of gestational age and gender slightly decreases. The value of the constant is reduced by 44 days and the other variables maintain the same approximate value. The goodness of fit, represented by the pseudo-*R* [35], is 0.1336 and 0.1394 for the first and second models, respectively.

## Discussion

The effectiveness of the DRG system is widely discussed in the literature. Investigating the factors that determine the variations in the resource use is one of the main goals from a price redefinition perspective. Our study shows a low DRG performance in terms of predicting the resource use for babies affected by RDS; this is explained by the great variability between groups. The main drivers of this variability are the characteristics at birth and the type of discharge. Gestational age and preterm death have a significant impact on LOS. The analysis of the VLBW variable allows measurement of the significance of the impact of birth weight on the LOS. For a child with a birth weight of less than 1,500 grams, the hospital stay increases by almost 15 days. The second model fits the data better than the first, as reflected by an increase in the pseudo-*R*^*2*^. The significance of the VLBW variable can also be seen in the cluster analysis results. Infants who are VLBWs with a birth weight lower than 1,020 grams should be included in a single group, while those ranging between 1,190 and 1,500 grams represent 50% of the points in a different cluster (often configuring outliers).

The VLBW group with a birth weight lower than 1,020 grams has the highest variability in the LOS and in the number of examinations. The reason for this great variability is explained by the fact that some infants who belong to this group died after birth. Death before discharge occurs more frequently in the VLBW group [36, 37]. Therefore, the similarity within this group is more attributable to the birth rather than clinical characteristics. In particular, the group that includes infants with the lowest birth weight is characterized by a mean LOS value of 75 days, while the cluster represented for the most part by term infants has the lowest mean LOS value of 12 days.

The DRG 386 includes both neonates with RDS and extremely immature infants. This implies that both preterm newborns and term infants with RDS should have a similar expected LOS and an equivalent resource consumption. However, from our results, it is evident that the admission cost for very preterm babies differs from that of term babies, even if they are discharged with the same DRG.

Clusters 1 and 2, where extremely immature infants are not considered, have a similar mean cost; they mainly include infants discharged with DRG 386, for which the reimbursement is almost thrice the effective cost. On the contrary, the mean cost for surviving patients in Cluster 4 is approximately twice the refund rate. Cluster 3 exhibits a mean cost that is quite similar to the reimbursement; however, if we consider only the VLBW included in this cluster, it results in a mean cost of €39,166, which makes the refund rate inappropriate again.

Additionally, considering a mean hospital day cost equal to €820, it follows that for the survival of VLBW babies, the reimbursement does not completely cover the inpatient cost, while the hospitalization for term or near term infants with RDS is more than rewarded by the reimbursement.

Diagnosis-related group 385 does not take into consideration birth weight or gestational age and does not distinguish between infants deceased a few hours after the birth or infants that died after several days in the NICU [17]. However, from the analysis, it is clear that for patients with RDS who die before 8 days, the reimbursement fully covers the costs. Therefore, our study clearly shows that the strong patient differences in LOS, number of examinations, birth weight, and gestational age indicate that DRG 386 needs to be improved and that a reclassification of neonatal admission with RDS may be a suitable solution. The actual reimbursement level for patients discharged with this DRG is underestimated on average. In fact, the related costs incurred by hospitals who accept a significant number of VLBW patients annually are much higher than their expected reimbursement. This might have undesirable effects on the Third Level NICUs which have to manage at least 50 VLBWs annually, in accordance with the State-Regions Agreement of the 16^th^ of December, 2010. Currently, we estimate a loss of €36,420 for every surviving baby with a birth weight lower than 1,170 grams.

In this situation, the reimbursement benefits hospitals that mainly treat term infants with RDS, for which the study shows a gain greater than €20,000 per patient. In addition, the profitable reimbursement rate for those cases discharged after a few days could lead to the “up-coding” phenomenon, whereby physicians could inappropriately designate RDS as the main or secondary diagnosis in order to obtain larger revenues.

According to our findings, four different tariffs might be set in order to reduce inefficiency and the undesirable effects that may occur given the high variability in preterm infants affected by RDS. The new tariff should equal the mean cost of hospitalization belonging to each cluster. Practically, the discriminant and observable variable that could be used by the policy maker to separate patients (and associate the correct reimbursement) among groups could be the "birth weight" variable, which is the only one that does not show any overlapping among groups. A new grouping based on the birth weight variable would also allow for the dichotomy between DRGs 385 and 386 to be overcome. In doing so, inefficiency in resource allocation and risk of strategic behavior by hospitals may be sensibly reduced. Notwithstanding, it should be noted that the risk of strategic behavior is very remote with reference to public hospitals (such as Gaslini Children’s Hospital) that do not pursue the profit maximization goal.

Our study presents several limitations. Firstly, although the Gaslini Hospital represents almost the totality of infants affected by the pathology in a region, the analysis takes into account patients hospitalized in only one NICU, thereby limiting the number of cases. Secondly, by considering only Italian costs, the research lacks comparison with others European NICUs. Thirdly, the study does not consider the lodging cost for the caregivers during the infants’ hospital stay and the possible deterioration in their quality of life, thereby underestimating the average cost from this perspective. The efficacy and robustness of this study could be improved by including a greater number of cases. Moreover, the cluster analysis approach may also be applied to other episodes of care, contributing to a revision of the current DRG classification. Despite the limitations of this study, our results may support the regulatory authority to improve DRG ability and explain resource use among complicated infants by further “birth weight adjustment,” thereby discouraging unfair hospital behaviors.

## Supporting information

S1 Fig Correlation between LOS, birth weight, gestational age, and number of examinations. (TIFF)

S2 Fig The WSS plot. (TIFF)

S3 Fig Dendogram. (TIFF)
