# Supplementary figures and images for: The effectiveness of the neonatal diagnosis-related group scheme
Source: PLoS One. 2020 Aug 12;15(8):e0236695. doi: 10.1371/journal.pone.0236695 (PMC7423098; doi:10.1371/journal.pone.0236695)

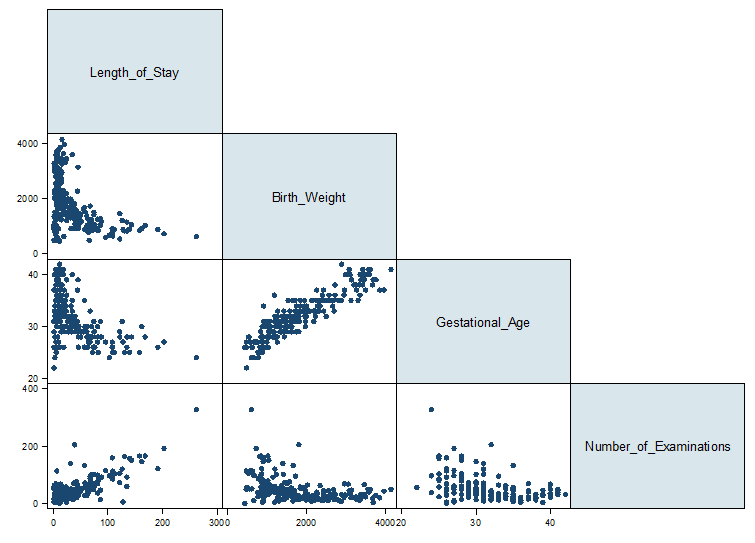

Supplement: S1 Fig — (TIFF) [file pone.0236695.s001.tiff]

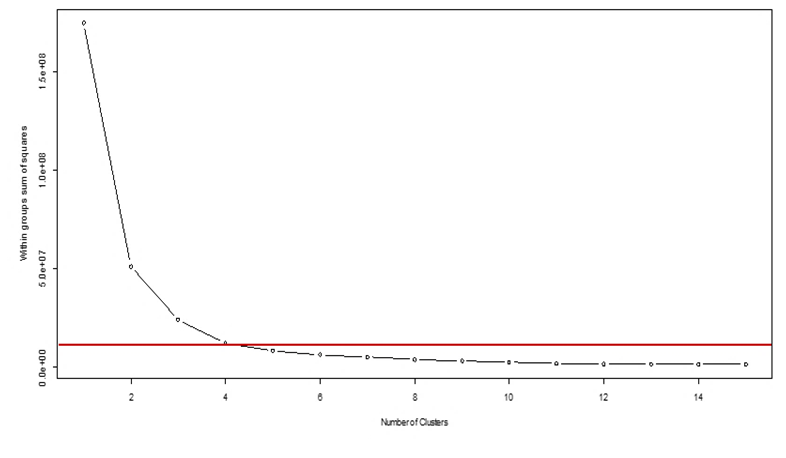

Supplement: S2 Fig — (TIFF) [file pone.0236695.s002.tiff]

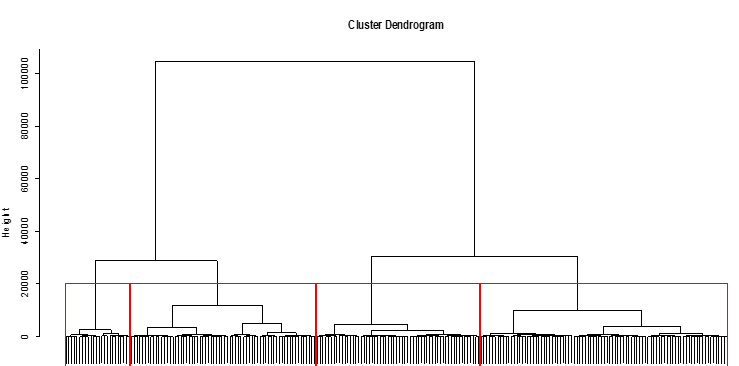

Supplement: S3 Fig — (TIFF) [file pone.0236695.s003.tiff]
